# Predictors Factors of Uncontrolled Masked Hypertension (MUCH) in Patients with Chronic Kidney Disease (CKD)

**DOI:** 10.3390/jcm14082663

**Published:** 2025-04-13

**Authors:** Roberto Santos Junior, Gabriel Fernandes Silva, Luciano Ferreira Drager, Andrea Pio-Abreu

**Affiliations:** 1 Faculty of Medicine, University of São Paulo (USP), São Paulo 01246-903, SP, Brazil; 2 Santa Casa de Tres Pontas, Três Pontas 37191-146, MG, Brazil

**Keywords:** masked uncontrolled hypertension, obesity, chronic kidney disease, predictors

## Abstract

**Background/Objectives**: Masked uncontrolled hypertension (MUCH) is a blood pressure phenotype prevalent among chronic kidney disease (CKD) patients. It has been associated with an elevated risk of cardiovascular morbidity and mortality. Identifying MUCH predictor factors in this population is crucial in facilitating anticipation of adverse outcomes and complications. **Methods**: For a period of 7 years (2017–2023), hypertensive patients presenting CKD and in-office normotension (<140/90 mmHg) were consecutively selected. After ambulatory blood pressure monitoring (ABPM), we classified the patients into controlled hypertension (CH) or MUCH. We used epidemiological, clinical, anthropometric, and laboratory data to develop a predictor model of the MUCH phenotype. **Results**: From 220 participants, 109 (49.5%) had MUCH (mean age: 60 ± 16 years; 45% men; 35% with obesity). Higher diastolic BP (DBP) values were observed in the MUCH group (72 vs. 75; *p* = 0.01). In contrast, a higher body mass index was observed in the CH group (26 vs. 28; *p* < 0.01), while elevated albuminuria was observed in the MUCH group (69 vs. 275; *p* < 0.01). After multivariate analysis, DBP ≥75 mmHg (Odds Ratio: 1.93, 95%CI 1.03–3.64; *p* = 0.04), BMI ≤25 Kg/m^2^ (Odds Ratio: 2.21, 95%CI 1.08–4.52; *p* = 0.03), and albuminuria ≥ 300 mg/g (Odds Ratio: 3.26, 95%CI 1.71–6.19; *p* < 0.01) were identified as predictors of MUCH phenotype **Conclusions**: MUCH is common in patients with arterial hypertension (AH) and CKD. DBP ≥ 75 mmHg, BMI ≤ 25 Kg/m^2^, and albuminuria ≥ 300 mg/g were predictors of MUCH in these patients.

## 1. Introduction

Masked uncontrolled hypertension (MUCH) and masked hypertension (MH) represent two distinct but related blood pressure (BP) phenotypes that can confound clinical management. MUCH is a blood pressure phenotype that occurs when in-office blood pressure (BP) values are standard (less than 140/90 mmHg) and out-of-office BP is elevated (24 h BP greater than 130/80 mmHg) for patients already under antihypertensive medication [1,2]. In contrast, MH refers to the phenomenon where individuals not on antihypertensive medication register regular BP measurements during routine office visits yet display hypertensive values during extended monitoring outside the clinical setting (such as with ambulatory blood pressure monitoring (ABP) or home measurements [3,4]). Both conditions are clinically significant because they may lead to underdiagnosis and undertreatment of hypertension.

The estimated prevalence of the phenotype ranges from 7% to 45%, and it is intrinsically associated with the sample studied [5,6]. In patients with chronic kidney disease (CKD), defined as an estimated glomerular filtration rate (eGFR) ≤ 60 mL/min/1.73 m^2^ or albuminuria ≥ 30 mg, the phenotype is even more frequent, reaching up to 56% [1,7,8,9]. In this context, it is noteworthy to emphasize the increasing prevalence of CKD patients worldwide in recent years, and associated hypertension is a pivotal factor in the progression of the disease [10].

The masked phenomenon has gained significant attention recently due to its significant association with adverse cardiovascular outcomes [1,6,9,11]. A meta-analysis carried out to compare various blood pressure phenotypes demonstrated that the masked groups (MH and MUCH) had higher mortality and cardiovascular outcomes compared to the control groups [12]. The same meta-analysis showed that the MUCH group had similar results to patients with sustained hypertension (characterized by uncontrolled BP in and out of the office) [12]. In addition, current studies demonstrated that the MUCH phenotype has worse outcomes related to target organ damage (TOD) compared to controlled hypertension (CH) patients, including reduced eGFR, increased albuminuria, vascular stiffness, and left ventricular hypertrophy (LVH) [9,13,14,15,16]. Similar results for cardiovascular events, mortality, and TOD were found in studies evaluating the effect of the masked phenotype in CKD patients [14].

The predictors of MUCH phenotype in CKD patients have yet to be thoroughly investigated. Considering the high prevalence of this blood pressure phenomenon, particularly among CKD patients, and the evidence of poorer clinical outcomes in this population, the identification of disease predictors is imperative. However, it is essential to refrain from a non-selective implementation of ABPM on a population scale, as this approach would result in escalated health service expenditures. The ability to promptly identify and intervene in this population could be pivotal in mitigating the progression of CKD, as well as cardiovascular events, morbidity, and mortality. Therefore, the present study was conducted in a real-world setting to identify potential predictors of MUCH phenotype in a sample of participants from a tertiary referral center specializing in treating hypertension and kidney disease.

## 2. Materials and Methods

### 2.1. Study Design and Participants

This retrospective cross-sectional observational study was conducted using electronic medical records. The study’s objective was to identify predictors of MUCH in a sample of patients with CKD. The research project was conducted following the principles of the Declaration of Helsinki. It was submitted for analysis by the Hospital das.

Clínicas Ethics Committee of the Faculty of Medicine of the University of São Paulo (FMUSP), number 3.008.827.

Patients were selected consecutively from a checklist of ABPM exams carried out between January 2017 and December 2023 at the FMUSP, Hospital das Clínicas, whose requests originated from the nephrology outpatient clinic of the aforementioned institution. This study selected patients with normal blood pressure (less than 140/90 mmHg) from a sample of patients evaluated at a medical office.

### 2.2. Inclusion and Exclusion Criteria

The study’s inclusion criteria were as follows: patients must be over the age of 18, diagnosed with CKD (glomerular filtration rate [GFR] 60 mL/min/1.73 m^2^ and/or albuminuria ≥ 30 mg/g), and under continuous antihypertensive medication. Patients under the age of 18, pregnant women, patients diagnosed with secondary hypertension, acute changes in eGFR in the last 3 months, changes to the antihypertensive regimen between in- and outpatient BP measurements, or those already undergoing renal replacement therapy (RRT) were excluded from the study. Furthermore, due to the ongoing pandemic during the period in which the ABPMs were collected, patients who tested positive for SARS-CoV-2 were excluded from the sample due to their inability to undergo the test at the time due to social isolation.

**Figure 1:**
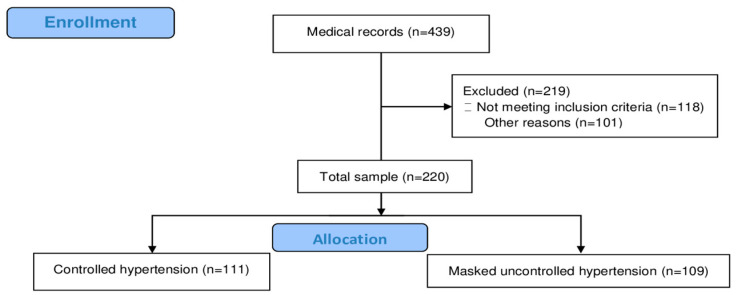
Flowchart of the cross-sectional study.

The flowchart of the study is seen in Figure 1.

### 2.3. Data Collection

A comprehensive data set was retrieved from electronic medical records, encompassing demographic, epidemiological, and clinical information documented up to the date of the clinical assessment where blood pressure (BP) was measured. The data set included gender, age, ethnicity, presence of diabetes mellitus (DM) and/or dyslipidemia, and the classes of antihypertensive medications being utilized. Anthropometric measurements were also recorded, including body mass index, waist circumference, hip circumference, and waist-to-hip ratio. Furthermore, laboratory results were retrieved, representing collection dates as close as possible to the time of the ABPM. The retrieved results encompassed serum creatinine, albumin/creatinine ratio of isolated urinary samples, and lipid profile.

### 2.4. Blood Pressure Measurement

In-office BP was measured using the oscillometric method with a Connex Spot Monitor device (Hillrom|Welch Allyn, Hill-Rom Allen Medical, MA, USA). Three measurements were obtained with an interval of 2–3 min between them, and the average of the last two was taken as the final value.

ABPM was taken every 20 min to measure out-of-office BP for 24 h. The OnTrak model from Spacelabs Healthcare (Spacelabes, WA, USA) and cuffs appropriate to each patient’s size were used. The 24 h average BP was used for diagnosis. The data relating to the ABPM report, including the averages during the daytime, nighttime, and 24 h periods, were also cataloged.

### 2.5. Classification of Participants

We collected the material on the date closest to the ABPMs for the creatinine and albuminuria variables. Participants were classified according to the KDIGO (2024) [10]. Laboratory data, including total cholesterol, LDL, HDL, triglycerides, and serum creatinine, were collected retrospectively from electronic medical records. All laboratory tests were performed in the institution’s certified clinical laboratory, following standardized protocols and quality control procedures. The most recent results obtained within three months before ABPM were considered for each participant. GFR was calculated from serum creatinine using the CKD-EPI formula (2021). Albuminuria was computed using the creatinine/albumin ratio of a single urine sample.

The BMI was calculated using the ratio between weight in kilograms and height (in square meters). Values and other anthropometric data were collected on the same day that office BP was accessed. Participants were classified according to WHO recommendations [17].

### 2.6. Phenotypical Categorization of Participants

Participants’ phenotypic categorization was based on office BP and the 24 h average recorded ABPM. According to the European Guideline of Cardiology, office BP is normal when <140/90 mmHg and ABPM > 130/80 mmHg [18]. Based on office BP values and ABPM averages, patients were classified into two groups: controlled hypertension (CH) when office BP and ABPM were normal and masked uncontrolled hypertension (MUCH) when office BP was normal, but ABPM was altered.

### 2.7. Statistical Analysis

The estimated sample size to be used was calculated using G*Power software, version 3.1. We assumed an expected prevalence of masked hypertension phenotype in patients with CKD of approximately 43%, based on the literature. For the calculation, we adopted an alpha error of 5% and a statistical power of 60%. The minimum required sample size was estimated at 193 participants. The final study sample included 220 participants, enough to ensure the statistical validity of the results obtained.

The data were analyzed using statistical software (R Commander version 3.2.1). For continuous quantitative variables, means and/or medians were used; for discrete quantitative variables, interquartile ranges were used. All quantitative variables were subjected to the Shapiro–Wilk normality test. When the variable was defined as parametric, Student’s *t-*test was used to compare the means when there were only two groups. If there were more than two groups, an ANOVA test was used. For non-parametric data, the Mann–Whitney test was used (when comparing only two groups), or the Kruskal-Walli’s test (when comparing more than two groups).

Non-quantitative variables were represented by frequency and percentage. The χ^2^ test or Fisher’s test was used to analyze the distribution of frequencies and compare the groups. Values of *p* < 0.05 were considered statistically significant. Finally, variables associated with the MUCH phenotype from univariate analysis, with a significance level of *p* < 0.2, were explored in a multivariate logistic regression analysis model to quantify the prediction variables.

## 3. Results

During the recruitment period, 773 candidates were initially selected. The final sample comprised 220 participants with complete data, with a mean age of 60 ± 16 years. Most of the participants were female (55%). Approximately 25% of the patients had been diagnosed with diabetes mellitus (DM), and the majority had dyslipidemia (Table 1).

The frequency of MUCH was 14%, and no statistical differences were found between the epidemiological variables and the CH group. However, the MUCH group had a higher in-office DBP than the control group, which was not observed for SBP (Table 1). As expected, when evaluating ABPM data, 24 h blood pressure averages were significantly higher in the MUCH group, with both daytime and nighttime measurements adding to the result. On the other hand, nocturnal dipping was absent (71%), and there was no difference between groups (Table 1).

The groups were similar regarding the number and quantity of antihypertensive medication classes used, but the MUCH group used fewer angiotensin-converting enzyme (ACE) inhibitors or angiotensin receptor blockers (ARBs) (64.2 vs. 77.5, *p* = 0.04; Table 1). When investigating the presence of resistant hypertension, this condition was observed in half of the total sample (41%), but there was no statistical difference between the groups (Table 1).

Table 2 represents the anthropometric data between the two groups. The overall sample had a mean BMI of 28.7 kg/m^2^ (±6.10). The masked phenotype had lower values (26 vs. 28 kg/m^2^, *p* < 0.01), although both were in the overweight classification range. When evaluating other parameters, no statistical difference was observed between groups.

For laboratory evaluation, the results are described in Table 3, emphasizing the higher albuminuria in the MUCH group (275 vs. 69, *p* < 0.01). There was no statistical difference between the other variables.

Comparing the participants according to the KDIGO classification for eGFR, no statistical difference was observed in the frequency of the MUCH phenotype across the staging periods. Nevertheless, a decline in nocturnal descent and increased BP values were observed during the nighttime as eGFR deteriorated (*p* = 0.05; Table 4).

In classifying patients based on albuminuria, a highly significant association of MUCH was observed for group A3, followed by A2 and A1 (34 vs. 47.1 vs. 65.3, *p* = 0.01, Table 4).

Phenotypic categorization of participants was based on office BP (<140/90 mmHg) and mean ABPM recorded over 24 h (>130/80 mmHg), according to the European Guideline of Cardiology [19]. 

**Figure 2:**
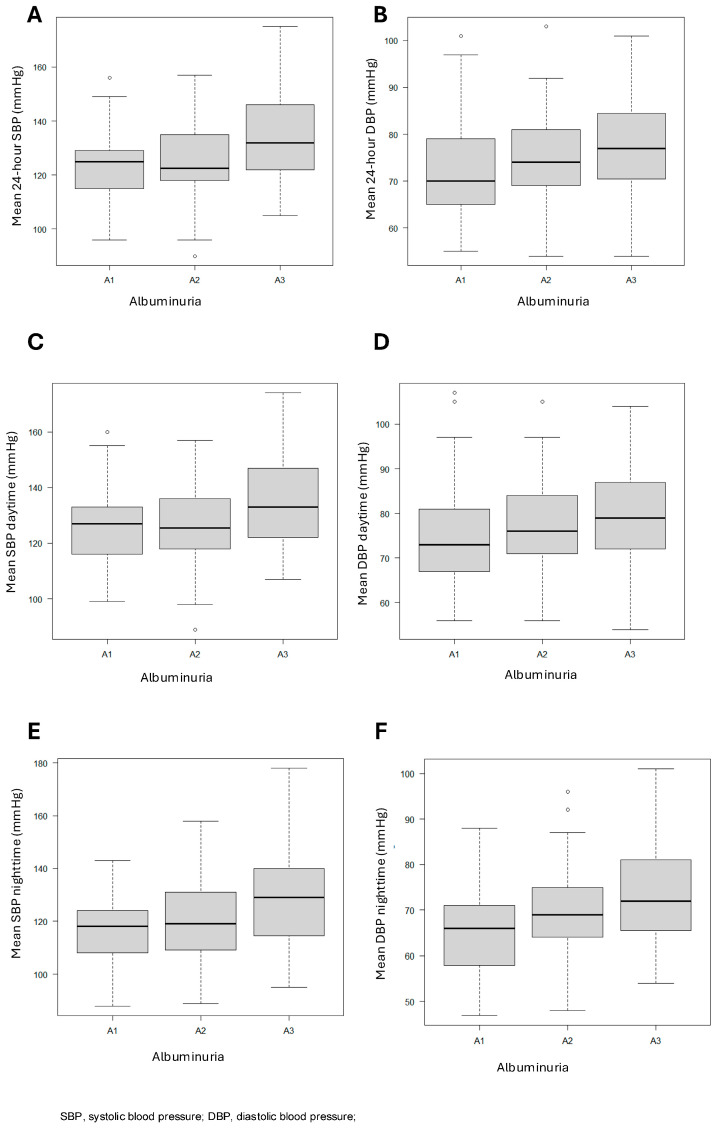
Blood pressure behavior during the 24 h period (**A**,**B**), daytime (**C**,**D**), and nighttime (**E**,**F**) among CKD groups, classified by KDIGO guidelines (2024) and albuminuria class. SBP, systolic blood pressure; DBP, diastolic blood pressure.

Comparison of blood pressure during the 24 h period (A and B), daytime period (C and D), and nighttime period (E and F) between CKD groups classified by KDIGO and according to albuminuria levels can be visualized in Figure 2.

Additionally, higher blood pressure levels, as described by ABPM, and diminished albuminuria values were observed (see Table 5).

The predictor variables for MUCH obtained from the multivariate analysis are presented in Table 6.

## 4. Discussion

Our study identified several key findings regarding the frequency and predictors of the MUCH phenotype in patients with CKD. Firstly, the MUCH phenotype was highly prevalent compared to the general population. Secondly, in-office diastolic blood pressure (DBP), albuminuria level, and BMI predicted MUCH phenotype in patients with CKD. However, it is noteworthy that differences in other epidemiological, anthropometric, and laboratory variables did not demonstrate a significant association with MUCH. 

The prevalence of MUCH was notably high in our sample, around 14%. Contrary to the present study’s findings, a range of values for CKD patients was reported. A study by Adamasco Cupisti [8], which had a similar profile of participants, found a prevalence of 7.9% for the MUCH phenotype. Another study [9] involving 333 non-dialyzed CKD patients revealed a prevalence of 32.8%. Higher rates were also observed [20], between 43 and 75%. The higher proportion of female patients in our cohort explains our lower prevalence. Previous studies have shown a stronger association between MUCH and male patients [21,22]. Variability in the prevalence across studies is due to differences in the parameters used for ABPM and the specific definitions of MUCH. A study by Coccina et al. [23] demonstrated that the prevalence of the MUCH phenotype varied based on ABPM criteria adopted, including whether it was based on a 24 h average, a daytime average, or a nighttime average.

### 4.1. Predictor Factors

Interestingly, while office systolic blood pressure (SBP) did not show a significant association with MUCH, previous studies have highlighted its role in the general population [22,24]. The study by Andalib [25] demonstrated that normal-high office SBP was a determinant of MH. Similarly, Jean-Michel Mallion [6] found an association between in-office SBP and the phenotype above 130 mmHg. In treated hypertensive patients, office DBP is more strongly associated with masked hypertension than SBP, particularly in those with metabolic comorbidities [26]. Although this population was not limited to patients with CKD, the findings highlight a broader relevance of DBP as a potential marker of inadequate BP control outside the clinical setting. Moreover, data from pediatric cohorts with CKD demonstrated that masked hypertension was often present despite apparently normal office BP, with DBP showing stronger correlations with ambulatory abnormalities [27]. The European Society of Cardiology and the European Society of Hypertension consider patients with even lower office BP (SBP greater than 120 mmHg and DBP greater than 70 mmHg) at risk of developing the masked phenotype [13,19].

Our study identified in-office diastolic blood pressure (DBP) as a significant predictor of MUCH, particularly at levels ≥75 mmHg. This is consistent with the American College of Cardiology recommendations to conduct ABPM in patients with office blood pressure ≥120/75 mmHg [28]. Other studies have also supported the predictive value of DBP for MUCH. Moreover, evidence from studies such as those by Shafi et al. [29] suggests that diastolic blood pressure control may be significant among CKD patients, potentially due to underlying pathophysiological differences related to vascular stiffness and volume status. These findings indicate that DBP is a potentially sensitive clinical marker of MUCH, especially in high-risk populations like CKD patients. Taken together, these findings emphasize the early detection of masked hypertension in this population, as it is strongly associated with increased cardiovascular risk and progression of renal disease [30].

Anthropometric measures were also evaluated as potential predictors of MUCH. While elevated anthropometric indices were noted, including a mean BMI of 28.7 kg/m^2^ and increased waist circumference, hip circumference, and waist-to-hip ratio, no statistically significant differences between conventional hypertension and the MUCH group were noted. Interestingly, despite the classification similarities, BMI was lower in the MUCH group, which contradicts prevailing literature. A recent study [31] found that being overweight and obese was associated with MUCH phenotype. This suggests that in-office BP control may be overestimated in this population. One hypothesis is that fluid overload or hypervolemia (a common condition in CKD patients) may not have been adequately factored into our anthropometric evaluation. Hypervolemia can independently influence BP readings, potentially obscuring associations with body weight indices. Studies have highlighted the impact of extracellular fluid volume on blood pressure regulation in CKD, urging future research to include direct measures of volume status to clarify these relationships [32].

The quantification of albuminuria was compared between the groups, and a higher value was observed in the MUCH phenotype. Increased albuminuria correlated with worse ABPM results and a higher frequency of MUCH. This is critical because albuminuria measurements are relatively simple to conduct in clinical settings compared to imaging methods evaluating vascular and cardiac status. Moreover, findings suggest that the masking mechanism may be directly related to renal dysfunction [30]. Given the established link between albuminuria and endothelial dysfunction and renal microvascular injury [33], our results indicate that simple laboratory measures can be surrogate markers for identifying patients at increased risk for adverse outcomes. Furthermore, research by Rahman et al. [34] supports the notion that abnormal ABPM patterns, alongside elevated albuminuria, could heighten cardiovascular event risk in CKD patients.

No differences in MUCH frequency were observed when categorizing patients by estimated glomerular filtration rate (eGFR) and KDIGO classification [10]. Similar data have been reported [9]. In our sample, however, the mean ABPM was higher the lower the eGFR, especially for SBP during nighttime, as noted before [35]. Clinically, these findings suggest using nighttime BP as an early warning indicator for worsening kidney function, which could aid clinicians in risk stratification and therapeutic intensification.

Other variables have been tested for predicting MUCH, with no significant association. These include the presence of DM, which, unlike our study, is currently shown to be an essential predictor of MUCH in most studies [5,25]. One potential reason for the observed outcome is that our sample population included a low proportion of patients with diabetes, probably influencing the accuracy of the prediction. In addition, it may be that the patients with diabetes in the sample were in reasonable control of their disease. Studies show that the relationship between MUCH and DM occurs mainly in patients with poorly controlled diabetes [36].

Our study found no statistically significant age difference between the groups, contradicting reports of a higher association of MUCH with males over 70 [6] or younger age groups (>40 years) [37]. It is hypothesized that age-related vascular changes might not be as prominent in our CKD population due to competing risk factors linked to renal dysfunction [38].

### 4.2. Limitations and Future Directions

This study’s cross-sectional design limits the ability to establish causal relationships between MUCH and associated factors. Longitudinal studies are needed to explore the temporal evolution of these phenotypes and determine whether interventions targeting predictors like fluid overload or nocturnal BP elevations can improve patient outcomes. Additionally, the real-world setting of our study precludes the assessment of damage to other target organs, such as left ventricular hypertrophy, as echocardiograms were not performed extensively on the study population, particularly during the ongoing pandemic. Prospective validation of the findings in a larger, multicenter cohort is necessary to confirm their reliability and ensure generalizability across diverse populations of patients with CKD. Such studies must also evaluate long-term clinical outcomes related to MUCH, including cardiovascular and renal events. Nonetheless, this limitation does not undermine our primary findings. 

## 5. Conclusions

Our findings underscore the importance of incorporating ABPM into routine CKD management, especially for patients with office DBP ≥75 mmHg. Identifying predictors, such as albuminuria and DBP, could help improve early detection of MUCH and inform personalized treatment strategies. However, more research is needed to standardize the definitions of MUCH, identify reliable predictors in diverse CKD populations, and explore the pathophysiological mechanisms underlying this phenotype. Future multicenter studies with larger, more diverse populations, advanced imaging techniques, and direct volume status assessments will be essential for refining the clinical management of CKD patients at risk for MUCH. 

## Figures and Tables

**Table 1:** Clinical and epidemiological characteristics of the sample population.

	All (*n* = 220)	CH (*n* = 111)	MUCH (*n* = 109)	*p*-Value
Age (years)	62 (49–73)	63 (51–76)	61 (48–70)	0.11
Gender M (%)	100 (45)	45 (40.5)	55 (50.5)	0.17
Race Nº (%)				
White	170 (77)	88 (79.3)	82 (75.2)	0.57
Non-white	50 (23)	23 (20.7)	27 (24.8)	
Diabetes Y (%)	53 (24)	28 (25.2)	25 (22.9)	0.81
Dyslipidemia Y (%)	168 (76)	92 (82.9)	76 (69.7)	0.03
No. of antihypertensives	3 (2–4)	3 (2–4)	3 (2–4)	0.69
No. of Antihypertensives Classes				
01 Class	41 (19)	16 (14.4)	25 (22.9)	0.4
02 Classes	48 (22)	27 (24.3)	21 (19.3)	
03 Classes	51 (23)	27 (24.3)	24 (22.0)	
04 or more Classes	80 (36)	41 (36.9)	39 (35.8)	
AH Resistant *n* (%)	91 (41)	41 (36.9)	50 (54.9)	0.22
Class of medication				
ACEI/ARB	156 (71)	86 (77.5)	70 (64.2)	0.04
CCB	126 (57)	62 (55.9)	64 (58.7)	0.77
BB	116 (53)	59 (53.2)	57 (52.3)	1
Thiazide	110 (50)	60 (54.1)	50 (45.9)	0.28
Office BP (mmHg)				
SBP	126 (117–131)	125 (113–131)	127 (120–131)	0.06
DBP	74 (66–79)	72 (64–87)	75 (67–82)	<0.01
ABPM (mmHg)				
24 h SBP	126 (116–136)	117 (111–123)	137 (131–175)	<0.01
24 h DBP	75 (68–82)	69 (65–74)	82 (76–88)	<0.01
SBP awake	122 (±14.82)	118 (±8.90)	140 (±10.95)	<0.01
DBP awake	77 (±10.57)	63 (±6.62)	75 (±9.51)	<0.01
SBP sleep	122 (±16.67)	111 (±9.81)	133 (±14.73)	<0.01
DBP sleep	69 (±10.32)	63 (±6.62)	75 (±9.51)	<0.01
ND Absent (%)	156 (71)	75 (67.6)	81 (74.3)	0.34

CH: controlled hypertension; MUCH, masked uncontrolled hypertension; M, male; No, number; Y, Yes; AH, arterial hypertension; ACEI, angiotensin-converting enzyme inhibitor; ARB, angiotensin receptor blocker; CCB, calcium channel blocker; BB, beta-blocker; BP, blood pressure; SBP, systolic blood pressure; DBP, diastolic blood pressure; ABPM, ambulatory blood pressure monitoring; ND, nocturnal decline; No, number.

**Table 2:** Comparison of anthropometric variables between groups.

	All (*n* = 220)	CH (*n* = 111)	MUCH (*n* = 109)	*p*-Value
BMI (Kg/m^2^)	27 (24–32)	28 (25–34)	26 (23–29)	<0.01
Waist (cm)				
M	101 (93–111)	103 (94–113)	101 (93–109)	0.33
F	99 (±14.20)	102 (±13.83)	95 (±13.83)	<0.01
Hip (cm)				
M	103 (96–110)	105 (96–112)	103 (97–109)	0.27
F	106 (98–115)	109 (98–118)	103 (98–111)	0.03
WHR				
M	0,98 (±0.05)	0.98 (±0.06)	0.98 (±0.05)	0.78
F	0,93 (0.88–0.97)	0.94 (0.90–0.97)	0.92 (0.85–0.97)	0.12

MUCH, masked uncontrolled hypertension; CH, controlled hypertension; BMI, body mass index; M, male; F, female; WHR, waist-to-hip ratio.

**Table 3:** Comparison of laboratory variables between the groups.

	All (*n* = 220)	CH (*n* = 111)	MUCH (*n* = 109)	*p*-Value
Creatinine (mg/dL)	1.65 (1.28–2.26)	1.57 (1.24–2.03)	1.75 (1.37–2.50)	0.07
GFR (CKD-EPI)	35 (26–49)	37 (27–51)	32 (25–49)	0.23
Albuminuria	121 (26–535)	69 (13–316)	275 (41–1,109)	<0.01
Total cholesterol (mg/dL)	172 (142–199)	175 (137–201)	171 (148–197)	0.78
LDL (mg/dL)	96 (71–120)	96 (72–118)	96 (68–121)	0.87
HDL (mg/dL)	47 (37–59)	47 (39–59)	46 (36–58)	0.46
Triglycerides (mg/dL)	132 (93–194)	132 (98–184)	142 (89–205)	0.87

MUCH, masked uncontrolled hypertension; CH, controlled hypertension; eGFR, estimated glomerular filtration rate; LDL, low-density lipoprotein; HDL, high-density lipoprotein.

**Table 4:** Blood pressure behavior according to the stages of the KDIGO classification (2024) for eGFR.

	G1 (13)	G2 (17)	G3a (51)	G3b (55)	G4 (70)	G5 (14)	*p*-Value
MUCH Phenotype *n* (%)	7 (53.8)	5 (29.4)	25 (49)	26 (47.3)	37 (52.9)	9 (64.3)	0.48
Absent ND *n* (%)	7 (53.8)	11 (64.7)	30 (58.8)	41 (74.5)	54 (77.1)	13 (92.9)	0.05
Clinic							
SBP	127	126	126	125	126	125	0.99
DBP	74	77	72	73	71	75	0.19
ABPM							
24 h SBP	126 (±10.22)	121 (±9.95)	127 (±13.11)	125 (±16.29)	128 (±15.13)	134 (±18.00)	0.17
24 h DBP	76 (±5.70)	76 (±6.74)	76 (±9.98)	74 (±10.67)	74 (±10.91)	79 (±10.75)	0.45
Daytime SBP	129 (±10.41)	123 (±9.84)	130 (±13.20)	126 (±16.62)	129 (±15.34)	134 (±17.92)	0.37
Daytime DBP	78.84 (±6.09)	79 (±7.31)	78 (±10.50)	76 (±10.97)	75 (±11.36)	80 (±11.84)	0.58
Night SBP	119 (±12.04)	115 (±12.79)	121 (±15.28)	118 (±17.53)	124 (±16.25)	136 (±20.04)	<0.01
Night DBP	68 (±6.91)	70 (±6.97)	69 (±10.25)	67 (±10.89)	69 (±10.73)	77 (±9.33)	0.04
No. Medications	2	3	3	3	3	3.5	0.04
Resistant AH No. (%)	3 (23.1)	10 (58.8)	15 (29.4)	25 (45.5)	30 (42.9)	8 (57.1)	0.11

MUCH, masked uncontrolled hypertension; No., number; ND, nocturnal descent; SBP, systolic blood pressure; DBP, diastolic blood pressure; ABPM, ambulatory blood pressure monitoring; AH, arterial hypertension.

**Table 5:** Blood pressure behavior for albuminuria according to the KDIGO (2024) classification.

	A1	A2	A3	*p*-Value
MUCH Phenotype *n* (%)	18 (34)	33 (47.1)	49 (65.3)	<0.01
Absent DN *n* (%)	34 (64.2)	52 (74.3)	58 (77.3)	0.24
Clinic				
SBP	124 (116–131)	126 (117–130)	127 (120–132)	0.35
DBP	73 (66–78)	73 (65–79)	74 (67–81)	0.55
ABPM				
24 h SBP	123 (±12.23)	125 (±14.51)	132 (±15.55)	<0.01
24 h DBP	72 (±11.06)	75 (±8.99)	78 (±10.18)	<0.01
Daytime SBP	125 (±13.17)	126 (±14.69)	134 (±15.21)	<0.01
Daytime DBP	74 (±11.96)	76 (±9.33)	79 (±10.54)	0.01
Night SBP	117 (±11.99)	120 (±15.68)	129 (±18.88)	<0.01
Night DBP	65 (±9.68)	69 (±9.36)	73 (±10.66)	<0.01
No. Medications	3 (2–4)	3 (2–4)	3 (2–4)	0.19
AH Resistant No. (%)	16 (30.2)	29 (41.4)	39 (52.0)	0.04

MUCH, masked uncontrolled hypertension; No, number; ND, nocturnal descent; SBP, systolic blood pressure; DBP, diastolic blood pressure; ABPM, ambulatory blood pressure monitoring; AH, arterial hypertension.

**Table 6:** Multivariate analysis using a logistic regression model to predict the MUCH phenotype.

	Odds Ratio	IC 95%	*p*-Value
BMI <25 Kg/m^2^	2.21	1.08–4.52	0.03
DBP ≥ 75 mmHg	1.93	1.03–3.64	0.04
Albuminuria ≥ 300 mg	3.26	1.71–6.19	<0.01

CI, confidence interval; BMI, body mass index; DBP, diastolic blood pressure.

## Data Availability

Data are available at the author’s request.
